# Metabolic management of glioblastoma multiforme using standard therapy together with a restricted ketogenic diet: Case Report

**DOI:** 10.1186/1743-7075-7-33

**Published:** 2010-04-22

**Authors:** Giulio Zuccoli, Norina Marcello, Anna Pisanello, Franco Servadei, Salvatore Vaccaro, Purna Mukherjee, Thomas N Seyfried

**Affiliations:** 1 Radiology Department, Arcispedale Santa Maria Nuova, Reggio E. 42100, Italy; 2 Neurology Department, Arcispedale Santa Maria Nuova, Reggio E. 42100, Italy; 3 Neurosurgery Department, Arcispedale Santa Maria Nuova, Reggio E. 42100, Italy; 4 Nutrition Department, Arcispedale Santa Maria Nuova, Reggio E. 42100, Italy; 5 Current address: Radiology Department University of Pittsburgh Medical Center, Children's Hospital of Pittsburgh, Pittsburgh, PA 15201, USA; 6 Biology Department, Boston College, Boston, MA 02467, USA

## Abstract

**Background:**

Management of glioblastoma multiforme (GBM) has been difficult using standard therapy (radiation with temozolomide chemotherapy). The ketogenic diet is used commonly to treat refractory epilepsy in children and, when administered in restricted amounts, can also target energy metabolism in brain tumors. We report the case of a 65-year-old woman who presented with progressive memory loss, chronic headaches, nausea, and a right hemisphere multi-centric tumor seen with magnetic resonance imaging (MRI). Following incomplete surgical resection, the patient was diagnosed with glioblastoma multiforme expressing hypermethylation of the *MGMT *gene promoter.

**Methods:**

Prior to initiation of the standard therapy, the patient conducted water-only therapeutic fasting and a restricted 4:1 (fat: carbohydrate + protein) ketogenic diet that delivered about 600 kcal/day. The patient also received the restricted ketogenic diet concomitantly during the standard treatment period. The diet was supplemented with vitamins and minerals. Steroid medication (dexamethasone) was removed during the course of the treatment. The patient was followed using MRI and positron emission tomography with fluoro-deoxy-glucose (FDG-PET).

**Results:**

After two months treatment, the patient's body weight was reduced by about 20% and no discernable brain tumor tissue was detected using either FDG-PET or MRI imaging. Biomarker changes showed reduced levels of blood glucose and elevated levels of urinary ketones. MRI evidence of tumor recurrence was found 10 weeks after suspension of strict diet therapy.

**Conclusion:**

This is the first report of confirmed GBM treated with standard therapy together with a restricted ketogenic diet. As rapid regression of GBM is rare in older patients following incomplete surgical resection and standard therapy alone, the response observed in this case could result in part from the action of the calorie restricted ketogenic diet. Further studies are needed to evaluate the efficacy of restricted ketogenic diets, administered alone or together with standard treatment, as a therapy for GBM and possibly other malignant brain tumors.

## Introduction

Glioblastoma multiforme (GBM) is the most malignant primary brain tumor in adults and children. Conventional GBM therapies are considered palliative, but rarely curative. Long-term progression free survival remains low for most GBM patients even after complete surgical excision, combined with the best available treatment [1]. Standard therapy for GBM includes surgery followed by concomitant radiation and/or chemotherapy. These procedures, however, extend median survival by only a few months beyond the no therapy option [2]. In general, survival is better for younger patients than for older patients and also for those patients with promoter hypermethylation of the O^6^-methylguanine methyltransferase (*MGMT*) gene [1,3]. Although numerous somatic mutations occur in GBM, no new therapies are yet available to exploit this information for enhanced patient survival [4]. The presence of numerous mutations in GBM tumor cells will, however, restrict metabolic flexibility thus enhancing susceptibility of the tumor cells to energy stress according to principles of evolutionary biology and metabolic control theory [5-7].

A high glycolytic rate with lactic acid production, resulting largely from impaired respiratory function, is a primary metabolic phenotype of GBM and of most cancers [5,6,8]. In contrast to normal brain cells, which evolved to metabolize ketone bodies for energy when glucose levels are reduced, most brain tumor cells are dependent on glycolysis for survival and are unable to metabolize ketone bodies for energy due to impaired mitochondrial function [9]. This metabolic deficiency allows the tumor cells to be metabolically isolated from normal cells. A strong dependence on glucose makes the tumor cells vulnerable to death using therapies that target glucose metabolism. The ketogenic diet, administered in restricted amounts, is ideally suited as a non-toxic metabolic therapy for managing malignant brain cancer because the diet naturally lowers circulating glucose levels while elevating levels of ketone bodies [9-11]. The ketogenic diet (KD) is a high fat, low carbohydrate diet that has been used for decades as an effective therapy for refractory seizures in children [6,12-14]. Otto and co-workers showed that a KD supplemented with omega-3 fatty acids and medium-chain triglycerides could delay growth of human gastric cancer cells in nude mice [15], while Freedland and co-workers have considered the role for low-carbohydrate KD in the management of prostate cancer [16]. The KD also has disease-modifying activity against neurodegenerative disorders and protective action against brain trauma and ischemic injuries [11,17-19]. Hence, the ketogenic diet administered in restricted amounts (R-KD) has potential as a non-toxic metabolic therapy against malignant brain cancer.

While dietary restriction and restricted ketogenic diet therapy is effective in targeting tumor energy metabolism and angiogenesis in experimental animal models [9,11,20-23], no studies have evaluated the efficacy of restricted ketogenic diets as a therapy for older patients with GBM. An earlier report, however, showed that the KD was effective in managing growth and enhancing progression free survival in two children with malignant brain tumors that were refractory to radiation and chemotherapy [10]. In this study, we used neuro-imaging to describe the response of a 65 year-old female GBM patient treated with standard therapy together with a restricted ketogenic diet.

## Case Report

A 65 year-old-female was admitted to Arcispedale Santa Maria Nuova, Reggio, Italy on December 5^th^, 2008 who presented with progressive memory loss, chronic headaches, and nausea. The symptoms were present, off-and-on, for about one month prior to diagnosis. Neurological examination showed mild left superior harm and facial paresis. The patient's family history included breast adenocarcinoma (mother), and ovarian carcinoma (sister). Past clinical history included post-pubertal headache, hysterectomy at the age of 37 years, chronic erosive gastritis and familial hypercholesterolemia controlled with lipid-lowering medication. The patient's blood pressure was 120/70, and within normal limits. Laboratory tests revealed an unremarkable complete blood count. Liver and renal functions were within normal limits. Blood biochemistry was essentially normal. Prior to therapeutic intervention, the patient's weight and height, were 64 kilograms (kg) (141 pounds) and 158 centimetres (62 inches), respectively. This height and weight related to an approximate body mass index (BMI) of 25.6 kg/m^2^.

**Figure 1:**
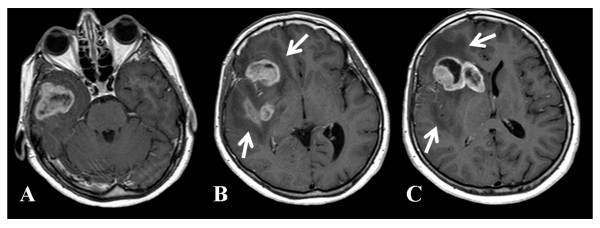
**MRI contrast enhanced images of a large multi-centric mass involving the right hemisphere pole**. (A) Temporal pole, (B) frontal operculum, insular lobe, posterior putamen, (C)  frontal operculum, head of caudate nucleus. Note the presence of peripheral edema (arrows).

On the day of admission, the patient underwent contrast-enhanced (contrast media: gadoteric acid, 0.2 ml/kg) magnetic resonance imaging (MRI), which disclosed a large multi-centric solid necrotic tumor in the right hemisphere (Figure 1). The tumor showed extensive infiltration of the right temporal pole, the insular lobe, the frontal operculum, the putamen, and head of the caudate nucleus. Avid contrast enhancement characterized the tumor, which was surrounded by extensive edema. A shift to the left of the midline structures was noted. The tumor also compressed the right frontal horn. An electroencephalogram demonstrated abnormality with a generalized slowing background and frequent delta bursts on the right frontotemporal region. Anti-inflammatory steroidal therapy (dexamethasone, 16 mg/day i.v.) and anti-epileptic therapy (Topiramate, 50 mg/2×/day and Clobazam, 50 mg/day) were commenced. On December 15^th^, the patient underwent right frontal temporal craniotomy involving partial excision of the temporal pole with incomplete debulking.

**Figure 2:**
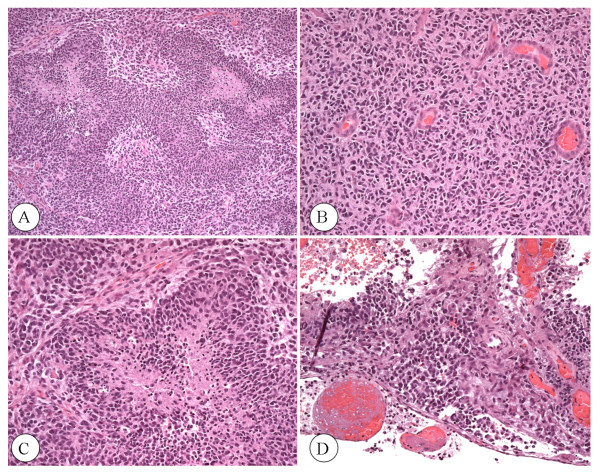
**Histopathological analysis of excised brain tumor tissue (H&E)**. A) Multiple zones of necrosis circumscribed by viable tissue (4 ×). B) Dense zone of small-cells with hyperchromatic nuclei and scant cytoplasm (10 ×). C) Zone of necrosis with dense pseudopalisades of small tumor cells at the periphery (20 ×). D) Tumor cell infiltration of the subpial zone of the cortex (20 ×).

The histopathological examination disclosed the patterns of GBM. Haematoxylin and eosin (H&E) analysis showed high cellularity, prominent vascularity, as well as areas of necrosis and hemorrhage. The tumor cells appeared poorly differentiated, hyperchromatic, pleomorphic, and displayed neoplastic pseudopalisades surrounding necrotic foci (Figure 2). Extensive subpial infiltration was also noted. The histological characteristics were typical for GBM (WHO grade IV) [24,25]. Methylation of the O^6^-methylguanine-DNA methyltransferase (MGMT) promoter was also detectable according to standard procedures [26]. The patient underwent computed tomography (CT) of the head during the immediate postoperative period, which showed an increasing edema-related shift to the left of the midline structures. During the immediate post-operative recovery period, the patient started a self-imposed water only fast (from December 16-17). The patient's average daily calorie intake, prior to fasting, was about 1700-1800 kcal/day. Blood glucose was 130 mg/dl while urine ketone levels were undetectable. After several days of modest food consumption, the patient again started a water-only fast on December 22. Blood glucose and urine ketones were monitored using a standard glucose kit and Keto-Stick kit (Ketur-Test^®^). After three days of fasting (ketosis induction period), ketones were raised to the high level of +++, while blood glucose was reduced to 60 mg/dl. At this time (December 24), the patient's body weight and body mass index was 58.0 kg (127.6 lbs) and 23.23 kg/m^2^, respectively. After the fast, a KD was administered in restricted amounts for 14 days (December 24 to January 7, 2009). This calorie restricted ketogenic diet (R-KD) delivered about 600 kcal/day in total and included 20 g of the KetoCal^® ^4:1 (fat/protein + carbohydrate) diet (SHS, International), 10 g medium chain triglyceride oil (MCT), 32 g protein, and 10 g carbohydrates. The diet contained a small amount of dietary fiber, and a total fat content of 42 g. The R-KD was supplemented with multivitamins including the B complex and minerals to maintain nutrient adequacy and to avoid metabolic abnormalities as previously described [27].

**Figure 3:**
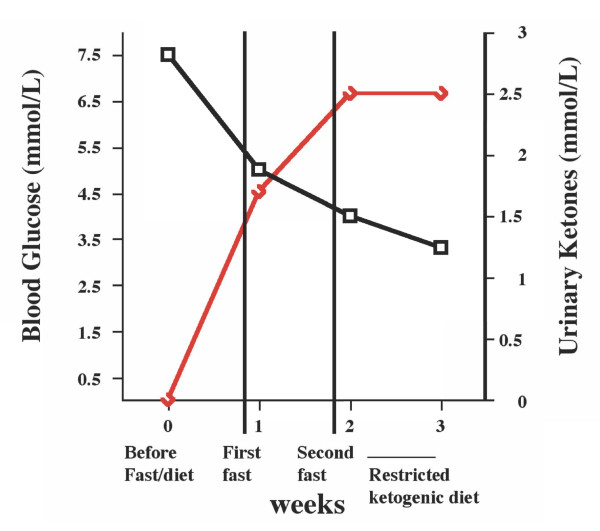
**Levels of circulating glucose (black line) and urinary ketones (red line) in the patient during the period from January 8 to February 7, 2009**. The values are within normal physiological ranges for people who maintain low calorie dieting [46].

After 14 days of the R-KD, the concomitant radiation plus chemotherapy (temozolomide) regimen was initiated on January 8, 2009, according to standard procedures [2]. All steroidal medication was terminated at this time. The patient's body weight was 55 kg (121 lbs) at the start of the standard treatment, which extended to February 17, 2009. On January 27, the patient developed a mild hyperuricemia of 6.2 mg/dl (normal values: 2.4-5.7 mg/dl). The plasma uric acid levels gradually increased reaching a maximum value of 10.9 mg/dl by February 7. Transient hyperuricemia can occur following implementation of ketogenic diets [28]. Allopurinol (100/mg/day) treatment was commenced to control the uric acid levels, which gradually returned within normal ranges. Due to the hyperuricemia the patient was gradually shifted to a calorie restricted non-ketogenic diet, which also delivered a total of about 600 kcal/day. This diet maintained low blood glucose levels and slightly elevated (++) urine ketone levels due to the low calorie content of the diet. The changes in circulating glucose and ketone levels during this period are shown in Figure 3. More comprehensive blood analysis was not conducted. It is important to mention that the patient did not experience hypoglycemia (blood glucose levels below 45 mg/dl) at any time during the course of fasting or ketogenic diet therapy.

**Figure 4:**
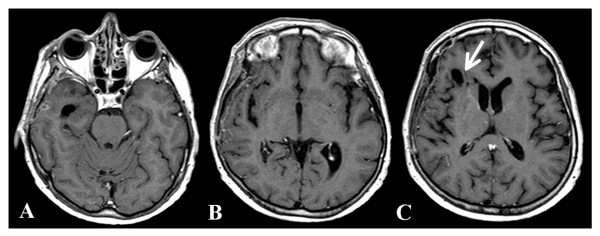
**Brain MRI taken a few days after ending the standard radiotherapy plus concomitant  temozolomide therapy together with KD-CR protocol.** No clear evidence of tumor tissue or  associated edema was seen. Arrow indicates porencephaly.

**Figure 5:**
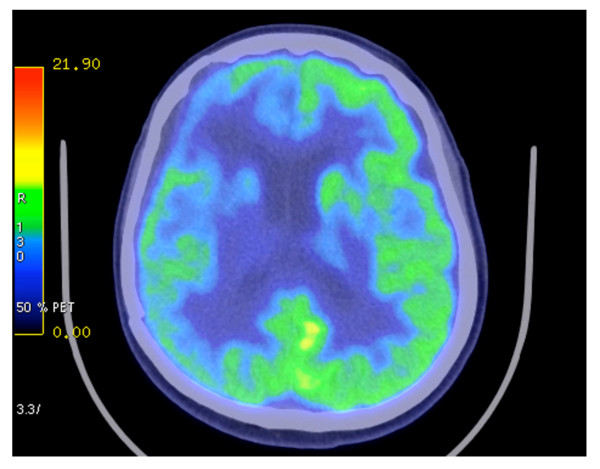
Positron emission tomography with fluoro-deoxy-glucose (FDG-PET) imaging  showing no evidence of recurrent tumor.

**Figure 6:**
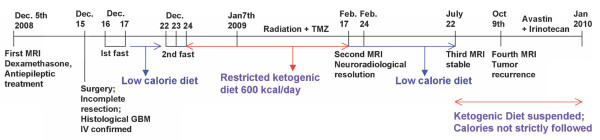
Timeline of clinical course with dates of dietary treatments, MRI, and PET

During the concomitant chemotherapy, laboratory tests revealed abnormalities in complete blood count to include leukocytes 2.86 ×1000/mm^3^, erythrocytes 3.56 million/mm^3^, hemoglobin 10.5 g/dl, and hematocrit 32%. The patient also developed lymphopenia 0.332 ×1000/mm^3^. The concomitant treatment was terminated on February 17^th^. One week later (February 24, 2009), the patient underwent an MRI. No evidence of either the tumor or the associated edema was apparent (Figure 4). Porencephaly was seen in the right frontal region at the tumor site. *Ex vacuum *enlargement of the right frontal horn and lack of mass effect represented indirect confirmation of tumor regression. *Restitutio ad Integrum *of the insular lobe, caudate nucleus, and putamen were noted with only minimal damage to the blood brain barrier (Figure 4). On March 3, the patient developed mild hypoproteinemia (5.1 g/dl). This was corrected by increasing dietary protein to about 7 g/day for one month, which returned protein levels to the normal range (6.4 gr/dl). On April 21, the patient underwent positron emission tomography with fluoro-deoxy-glucose (FDG-PET), which included delayed acquisition. No evidence of recurrent disease was detected (Figure 5). An MRI, performed on July 22, was stable over the comparison time from February 24 with no clear evidence of disease recurrence. At that time, the patient weighed 50 kg (110 pounds, BMI 20.0 kg/m^2^), was in good general health, and had no neurological complications. The patient's Karnofsky performance status was at 100% during the course of the diet. Caloric intake was not strictly followed after July 22. An MRI performed on October 9, 2008 showed tumor recurrence. The patient was then treated with CPT11 (Irinotecan) and bevacizumab (Avastin) therapy. A schematic diagram showing the clinical time course of dietary treatments with dates of MRI and PET is presented in Figure 6.

## Discussion

In this case report we describe the management of a highly invasive multi-centric GBM in an older patient following partial tumor resection and treatment with a combination of standard therapy, fasting, and a R-KD. The patient's response to this therapeutic approach was unusual, as no prior reports have appeared to our knowledge describing regression of GBM within 2.5 months from the time of diagnosis in either younger (< 50 yrs) or older (> 50 yrs) patients using standard radiation and temozolomide therapy alone. Although the patient in this study expressed hypermethylation of the *MGMT *gene promoter, which enhances the therapeutic action of temozolomide and is prognostic for increased survival [26], no prior cases of rapid GBM regression have been reported in patients with the MGMT hypermethylation phenotype to our knowledge. Temozolomide is an oral alkylating agent that damages DNA and is used as first and second line GBM treatment [2,26,29]. Continuous temozolomide administration depletes O^6^-methylguanine-DNA methyltransferase, which is required for repairing DNA damage. Based on the MRI and PET-CT data, we speculate that the combined conventional and metabolic approach to GBM management in this patient enhanced early MGMT related cytotoxicity and apoptosis. Further studies in additional patients will be needed to support this hypothesis.

The response of the GBM in this older female patient to the therapeutic action of the R-KD was similar to that reported previously in children with malignant brain tumors treated with a medium-chain triglyceride ketogenic diet [6,10]. High dosage steroid medication for brain cancer patients increases gluconeogenesis and blood glucose levels while enhancing apoptosis resistance in tumor cells [6,30,31]. We eliminated dexamethasone administration soon after surgery in our patient, as calorie restriction and the R-KD can also target inflammation without elevating blood glucose levels [6,30]. We consider that dexamethasone, which induces hyperglycemia, could antagonize metabolic management of GBM. While the findings in our patient are anecdotal, we cannot exclude the possibility that the management observed was related to the elimination of steroids and the combined action of standard therapy with early implementation of a novel metabolic therapy involving fasting, a R-KD, and calorie restriction.

It is well documented that brain tumor growth in mice is dependent to a large extent on circulating levels of glucose [11,32]. The same phenomenon also appears to be the case for human brain cancer patients, as reduced survival is associated with high blood glucose levels [33-35]. Glucose levels in brain are correlated with glucose levels in blood, but glucose concentration is lower in brain than in blood [36]. High circulating glucose levels accelerate brain tumor growth and angiogenesis while also preventing apoptosis through activation of the IGF-1/PI3K/Akt/Hif-1a signalling pathways [11,21]. Reductions in circulating glucose levels reverse these processes leading to reduced tumor growth [5,7,9,21]. Studies in mice also show that the therapeutic action of R-KD can be enhanced when combined with the glycolysis inhibitor, 2-deoxyglucose, for management of malignant astrocytoma [37]. Hence, pharmacological inhibition of glycolysis, while maintaining low circulating glucose levels (within normal physiological ranges), could be therapeutically beneficial to brain cancer patients.

Besides reducing inflammation, ketone bodies provide an alternative metabolic fuel for normal brain cells when glucose levels are reduced, and thus protect normal brain cells from the energy stress of reduced glucose levels [6,7]. Although long-term use of ketogenic diets can sometimes produce adverse effects (gastrointestinal disturbances, renal stones, etc) [12,38], these are generally mild and can be significantly reduced if the diet is consumed in restricted amounts. No adverse effects on neurological or physiological function were observed during the course of the metabolic therapy in our patient. Previous studies in rats also showed that calorie restriction could reduce inflammation while improving macrophage function suggesting improvements in some aspects of host immunity [39]. It is therefore unlikely that the R-KD would compromise host immune function. As long as the KD is consumed in restricted amounts, there should be no adverse effects on normal physiological functions.

In contrast to normal brain cells, the tumor cells are largely unable to metabolize ketone bodies for energy due to mitochondrial defects [9,30,40]. Moreover, recent studies in a variety of cultured human tumors cells show that ketone bodies inhibit the viability of tumor cells, but not of normal cells, suggesting that ketone bodies could inhibit tumor cell growth through multiple mechanisms [41,42]. The numerous mutations expressed in the tumor cells reduce metabolic flexibility thus rendering the tumor cells vulnerable to the therapeutic action of the R-KD [5,7].

The findings from our patient suggest that therapies, which lower blood glucose levels while elevating ketone body levels, could be an effective non-toxic therapy for increasing progression free survival in patients with malignant brain tumors. While our patient did not maintain blood glucose levels considered maximal for therapeutic efficacy (55-65 mg/dl) [7], the levels were reduced to low normal range. It is important to mention that measurement of urinary ketones is less predictive of physiological ketosis than is measurement of blood ketones. Although blood ketone levels can be correlated with urinary ketone levels, the correlation is not always accurate [43,44]. Consequently, measurements of blood ketone levels are recommended for future studies of ketosis state and therapeutic success in brain cancer patients. The combined action of reduced blood glucose together with elevated blood ketone levels could provide an effective complimentary or alternative non-toxic therapy for persons with malignant brain cancer. Clinical trials for ketogenic diet therapy for brain cancer management could be designed in a similar manner to those previously used for the management of epilepsy [45].

## Conclusion

This case report is remarkable for a number of reasons. First, this patient demonstrated that the R-KD was well tolerated suggesting that this diet could be an effective adjuvant treatment for GBM in adults. Second, the response of the GBM in this patient after standard treatment alone would be unlikely, further suggesting a role for targeting energy metabolism as part of the management strategy. Third, suppression of edema was achieved during the concomitant radiation and chemotherapy treatment without steroids, supporting the anti-inflammatory activity of calorie restriction and the R-KD. Finally, an established mechanism of action based on defective mitochondrial function in tumor cells can account for the potential therapeutic efficacy of the R-KD. In conclusion, further studies are required to determine the therapeutic significance of R-KD for general management of human GBM.

## Competing interests

The authors declare that they have no competing interests.

## Authors' contributions

GZ carried out all described methods and drafted the manuscript. NM, AP, FS and SV participated in the patient management, clinical data collection, and analysis. PM was responsible for the analysis and presentation of the data in Figures 3 and 6 and helped prepare the manuscript. TNS participated in study design, data analysis, and helped prepare the manuscript. All authors read and approved the final manuscript.
